# microRNA‐124 inhibits stem‐like properties and enhances radiosensitivity in nasopharyngeal carcinoma cells via direct repression of expression of JAMA

**DOI:** 10.1111/jcmm.15177

**Published:** 2020-07-17

**Authors:** Yunhong Tian, Yunming Tian, Yinuo Tu, Guoqian Zhang, Xing Zeng, Jie Lin, Meiling Ai, Zixu Mao, Ronghui Zheng, Yawei Yuan

**Affiliations:** ^1^ State Key Laboratory of Respiratory Disease Affiliated Cancer Hospital & Institute of Guangzhou Medical University Guangzhou China; ^2^ Department of Radiation Oncology Hui Zhou Municipal Central Hospital Huizhou China; ^3^ Department of Pharmacology and Chemical Biology School of Medicine Emory University Atlanta GA USA

**Keywords:** junctional adhesion molecule A, microRNA‐124, nasopharyngeal carcinoma, stem‐like properties

## Abstract

Cancer stem cells (CSCs) are a source of tumour recurrence in patients with nasopharyngeal carcinoma (NPC); however, the function of microRNA‐124 (miR‐124) in NPC CSCs has not been clearly defined. In this study, we investigated the role of miR‐124 in NPC CSCs. qRT‐PCR was performed to measure miR‐124 expression in NPC tissues and cell lines and the effects of miR‐124 on stem‐like properties and radiosensitivity of NPC cells measured. Luciferase reporter assays and rescue experiments were used to investigate the interaction of miR‐124 with the 3′UTR of junctional adhesion molecule A (JAMA). Finally, we examined the effects of miR‐124 in an animal model and clinical samples. Down‐regulation of miR‐124 was detected in cancer tissues and was inversely associated with tumour stage and lymph node metastasis. Overexpression of miR‐124 inhibited stemness properties and enhanced radiosensitivity of NPC cells in vitro and in vivo via targeting JAMA. Up‐regulation of miR‐124 was correlated with superior overall survival of patients with NPC. Our study demonstrates that miR‐124 can inhibit stem‐like properties and enhance radiosensitivity by directly targeting JAMA in NPC. These findings provide novel insights into the molecular mechanisms underlying therapy failure in NPC.

## 1 INTRODUCTION

Nasopharyngeal carcinoma (NPC) is a unique epithelial malignancy arising from the superior aspect of the pharyngeal mucosal space and is most common in southern China (incidence, 25‐50 per 100 000) and South‐East Asia.^1^ Although standard combination of radiotherapy and chemotherapy has improved treatment efficacy, local recurrence is a major cause of mortality and morbidity in the advanced stages of disease.^2^ Therefore, it is important to investigate the mechanisms underlying tumour recurrence and resistance to therapy in patients with NPC.

Cancer stem cells (CSCs) constitute a small subpopulation of cancer cells within tumours with stem‐like properties. CSCs can self‐renew, generate differentiated daughter cells, give rise to heterogeneous tumour tissue and are present in multiple different human malignancies.^3^ Wang et al showed that side population (SP) cells sorted from NPC cell lines are a source of CSCs, based on their 20‐fold greater ability to form tumours, relative to non‐SP cells.^4^ CSC markers include aldehyde dehydrogenase 1 (ALDH1), ATP‐binding cassette subfamily G member 2 (ABCG2), SRY (sex‐determining region Y)‐box2 (SOX2) and OCT4,^5^ and CSCs can form tumorspheres in serum‐free culture, because of their ability to self‐renew.^6^ CSCs are widely accepted as potential mediators of therapeutic resistance and considered targets for anticancer treatments.^7^, ^8^, ^9^ Above all, CSCs are a source of tumour recurrence and resistance to treatment.

microRNAs (miRNAs) are small (20‐22 nucleotide) non‐coding RNA molecules that function as post‐transcriptional regulators by negatively modulating the stability or translational efficiency of their target mRNAs.^10^ miRNAs have fundamental roles in controlling the fate of CSCs, and dysregulation of miRNAs is implicated in tumorigenesis.^11^, ^12^ The stem cell‐related miRNA, miR‐124, is highly conserved across diverse species and was originally identified as an abundantly expressed in the central nervous system.^13^ miR‐124 acts as a tumour suppressor in several human cancers, including glioblastoma, as it inhibits tumour cell proliferation and invasion.^14^ Moreover, miR‐124 plays a crucial role during the transition from neuronal stem cells to neuronal progenitors.^15^ Overall, evidence suggests that miR‐124 could be important for the maintenance of stem‐like properties of cancer cells and neural cells.

Junctional adhesion molecule A (JAMA), also known as JAM‐1 and F11R, belongs to a family of cell adhesion molecules that localize to intercellular junctions. JAMA has key roles in tight junction assembly, epithelial barrier function and epithelial‐mesenchymal transition (EMT).^16^, ^17^ Moreover, JAMA regulates epithelial and endothelial stem‐like behaviour^18^, ^19^; for example, Sugano et al confirmed that JAMA was expressed at high levels in the enriched haematopoietic stem cell (HSC) fraction, whereas multi‐lineage colony‐forming activity in JAMA‐positive cells is higher than that in JAMA‐negative cells.^20^ Taken together, the evidence strongly supports the function of JAMA in regulating stem‐like behaviour of CSCs.

Cancer stem cells can be regulated by aberrant miRNA expression, and miR‐124 and JAMA are involved in the development of stem‐like traits; however, the role of miR‐124 in NPC CSCs has not been clearly defined. In the current study, we investigated the role of miR‐124 in NPC CSCs. We found that miR‐124 expression was down‐regulated in cancer compared with normal tissues and inversely associated with tumour stage and lymph node metastasis. Overexpression of miR‐124 inhibited NPC stem‐like properties in vitro and in vivo, whereas down‐regulation of miR‐124 promoted NPC cell stemness. Furthermore, we showed that miR‐124 control stemness through direct regulation of JAMA expression. Specifically, our study demonstrates that miR‐124 can inhibit stem‐like properties and enhance radiosensitivity by directly targeting JAMA in NPC, suggesting that miR‐124 has potential value for diagnosis and as a therapeutic target in NPC.

## 2 MATERIALS AND METHODS

### 2.1 Cell culture and reagents

The human NPC cell lines, CNE1, CNE2, HONE1 and SUNE1, were originally purchased from the American Type Culture Collection (ATCC). CNE2 and HONE1 cell lines tested negative for mycoplasma in 2012. The authenticity of cell lines in our study was verified using the DNA finger printing method.^21^ Cells were maintained in RPMI 1640 medium supplemented with 10% foetal bovine serum (FBS; HyClone) and antibiotics, according to ATCC cell culture protocols. The immortalized cell line, NP69, maintained in our laboratory, was a kind gift from Dr Tsao (Cancer Center, Hong Kong University, Hong Kong). NP69 cells were cultured in keratinocyte serum‐free medium (K‐SFM) (Gibco). HEK293T cells were originally purchased from ATCC and maintained in Dulbecco's modified Eagle's medium (DMEM, Gibco) supplemented with 10% FBS (HyClone). All cells were incubated at 37°C in a humidified atmosphere with 5% CO_2_. microRNA‐124 mimics, negative control (NC) and inhibitors were purchased from RiboBio.

### 2.2 Tumorsphere formation assay

Single‐cell suspensions (2 × 10^3^ cells per well) were plated on 6‐well ultra‐low attachment plates (Corning Inc) in serum‐free DMEM/F‐12 (HyClone), supplemented with 10 ng/mL basic fibroblast growth factor (PeproTech), 20 ng/mL epidermal growth factor (Sigma‐Aldrich), 0.4% bovine serum albumin (Sigma‐Aldrich) and 2% B27 supplement (Invitrogen). Cultures were fed weekly and passaged every 2 weeks. During passaging, tumorspheres were harvested. The number of tumorspheres formed (diameter ≥40 μm) was counted under a microscope after 14 days of culture.

### 2.3 Flow cytometry analysis for SP

Once cells had reached a logarithmic growth phase, they were analysed by flow cytometry. Cells were digested and washed twice with calcium‐ and magnesium‐free PBS. Cells were then stained with 5 mg/mL Hoechst 33342 (Sigma‐Aldrich) in RPMI 1640 medium (supplemented with 2% FBS) at 37°C for 90 minutes in the dark, with mixing at intervals. Subsequently, cells were washed with PBS, and 1 μg/mL propidium iodide was (Sigma‐Aldrich) added; cells were kept at 4°C in the dark before sorting by fluorescence‐activated cell sorting (BD Biosciences). A subset of the cells was incubated with 50 μmol/L verapamil for 30 minutes at 37°C before adding Hoechst 33342 to determine the region that contained putative SP cells.

### 2.4 Total RNA extraction and quantitative reverse‐transcriptase polymerase chain reaction (qRT‐PCR)

Total RNA was extracted using TRIzol reagent (Invitrogen), as described in our previous report.^22^ For miRNA analysis, 1 μL of stem‐loop RT primer (1 μmol/L) and 2 μg of total RNA were used for first‐strand cDNA synthesis with an iScript™ cDNA Synthesis Kit (Bio‐Rad). For mRNA analysis, total RNA (4 μg) was transcribed into cDNA using the iScript™ cDNA Synthesis Kit (Bio‐Rad). Quantitative RT‐PCR was performed on an Mx3005P qPCR System (Agilent Technologies) using an iQ™ SYBR^®^ Green Supermix Kit (Bio‐Rad). The thermocycler programme for qPCR was as follows: initial denaturation at 95°C for 10 seconds, followed by 40 cycles of 10 seconds at 95°C and 20 seconds at 55°C. The threshold cycle (*Ct* value) was recorded. Quantitative RT‐PCR assays were repeated at least three times to ensure statistical rigour. Finally, mRNA and miRNA expression levels were calculated from three independent biological replicates. Fold changes in gene expression were calculated by relative quantification using the 2^△△^ *C*_T_ method.

### 2.5 Plasmid construction, retroviral infection, transfection and luciferase reporter assay

The construct, pWPI‐JAMA, empty pWPI vectors and JAMA shRNA were generated as previously described.^17^ Human miR‐124 was PCR‐amplified from genomic DNA and cloned into a pMSCV‐puro retroviral vector. pMSCV‐miR‐124 was cotransfected into HEK293T cells with a packaging plasmid. Retrovirus‐containing cell culture supernatants were harvested every 24 hours until 72 hours after transfection. Cells were infected with the virus in the presence of 8 μg/mL of polybrene (Sigma‐Aldrich). After 48 hours of infection, cells were treated with puromycin for selection and antibiotic‐resistant cells pooled for subsequent analyses.

Putative miR‐124 target genes were predicted using two miRNA target databases PicTar (http://www.pictar.org/) and TargetScan (http://www.targetscan.org/). The 3′ untranslated region (UTR) region of the human JAMA gene was PCR‐amplified from genomic DNA and cloned into the psiCHECK‐2 dual‐luciferase reporter plasmid (Promega). Luciferase reporter assays using the 3′UTR sequence were performed as previously described.^22^ Transfection of plasmids or oligonucleotides was conducted using Lipofectamine 2000 reagent (Invitrogen), according to the manufacturer's instructions.

### 2.6 Caspase‐3 activity and colony formation assays

Caspase‐3 activity was measured using a caspase‐3 Activity Assay Kit (Beyotime Institute of Biotechnology, Nangjing, Jiangsu province, China), as described in our previous report.^23^ Briefly, cells transfected with mimic or inhibitor were incubated for 12 hours following irradiation at a dose of 4 Gy. Cells were lysed with lysis buffer (100 μL per 2 × 10^6^ cells) for 15 minutes on ice and washed with D‐Hank's medium. Cell extracts mixed with Ac‐DEVD‐pNA substrate were incubated at 37°C for 2 hours. Extracts were analysed by colorimetric measurement of the p‐nitroanilide product at 405 nm and results normalized to those of NC assays, to determine the fold change in caspase‐3 activity. For colony formation assays, cells transfected with mimic, NC or inhibitor were incubated for 12 hours. Cells exposed to 0, 2, 4, 6 or 8 Gy of 6 MeV X‐ray radiation at room temperature were seeded at 500, 1000, 2000, 4000 or 8000 cells, respectively, in 100‐mm culture plates. Following irradiation, cells were incubated for 12 days to allow colony formation and surviving fractions (SFs) calculated. Dose‐survival curves were fitted based on the single‐hit multi‐target theory formula, as detailed in our previous report.^23^

### 2.7 Mouse xenograft assay

Female BALB/c nude mice (4‐6 weeks old) were purchased from the Model Animal Research Center of Nanjing University (Nangjing, Jiangsu Province, China). All mouse experiments were approved by the animal care and use committee of the Affiliated Cancer Hospital & Institute of Guangzhou Medical University (Guangzhou). To measure the effects of miR‐124 on stemness properties, each mouse was injected in the flank with cells stably transfected with vector or miR‐124 at a total of 500, 5000, 10^4^ or 10^6^ cells. Tumour volumes were measured every 7 days for 42 days, and mice were killed on day 42. To assess radiosensitivity, animals were injected subcutaneously into the right hindlimb with cells stably transfected with vector or miR‐124 (5 × 10^6^ cells/100 μL). After 2 weeks, mice whose tumour volumes reached approximately 200 mm^3^ were divided into four groups. For irradiation group, mice were irradiated with 6 MV X‐ray at a total dose of 20 Gy (2 Gy/day × 10 fractions). Tumour volumes were calculated according to the following formula: volume (mm^3^) = length × width^2^/2.

### 2.8 Clinical samples and immunohistochemistry

All samples were collected from the Affiliated Cancer Hospital & Institute of Guangzhou Medical University (Guangzhou) and subjected to histopathological examination. A total of 35 NPC and 15 non‐cancer nasopharyngitis (NP) tissues specimens were collected for qRT‐PCR. For overall survival (OS) analysis, another 121 samples from patients with NPC were collected, as previously described.^24^ To ensure that each tissue contained >80% homogeneous cancer cells in its cross‐sectional area, haematoxylin‐and‐eosin–stained sections matched for each NPC tissue were examined. JAMA expression was measured, as previously described, by immunohistochemistry and Western blotting analysis.^17^ All patients involved in this study signed an informed consent form. This research was approved by the medical ethics committee of the Affiliated Cancer Hospital & Institute of Guangzhou Medical University (Guangzhou).

### 2.9 Western blotting

Protein extracts from cells or tumour tissues were mixed with loading buffer, heated at 70°C for 10 minutes, separated on SDS‐PAGE gels and transferred to polyvinylidene fluoride (Millipore) membranes. Membranes were blocked for 2 hours in 5% bovine serum albumin and incubated overnight at 4°C with SP rabbit polyclonal antibodies against SOX2 (H‐65; sc‐20088, Santa Cruz, 1/1000), OCT4 (H‐134; sc‐9081, Santa Cruz, 1/1000), ABCG2 (B‐25; sc‐130933, Santa Cruz, 1/1000), JAMA (H202‐106; sc‐59845, Santa Cruz, 1/1000), Akt (H‐136; sc‐8312, Santa Cruz, 1/1000), ALDH1 (ab52492, Abcam, 1/1000), ABCG2 (ab207732, Abcam, 1/1000) and GAPDH (FL‐335; sc‐25788, Santa Cruz, 1/1000). Membranes were then incubated with horseradish peroxidase (HRP)–conjugated secondary antibody (sc‐2004, Santa Cruz, 1:1000) for 1 hour at room temperature. Finally, bands were visualized using enhanced chemiluminescence (Thermo Scientific Pierce).

### 2.10 Statistical analysis

Statistical analyses were performed with SPSS statistical package version 15.0. In vitro experiments were repeated three times and data are presented as the mean ± SE, unless otherwise noted. Statistical differences among groups were assessed using one‐way analysis of variance (ANOVA). Mouse xenografts were analysed by the single‐hit Poisson model, using the extreme limiting dilution analysis (ELDA) tool (http://bioinf.wehi.edu.au/software/elda).^25^ Kaplan‐Meier analysis and the log‐rank test were used to evaluate differences in OS rates between groups. *P*‐values <.05 were considered statistically significant.

## 3 RESULTS

### 3.1 miR‐124 expression is down‐regulated in NPC cell lines and tumour tissues, and inversely correlated with clinical stage

**FIGURE 1:**
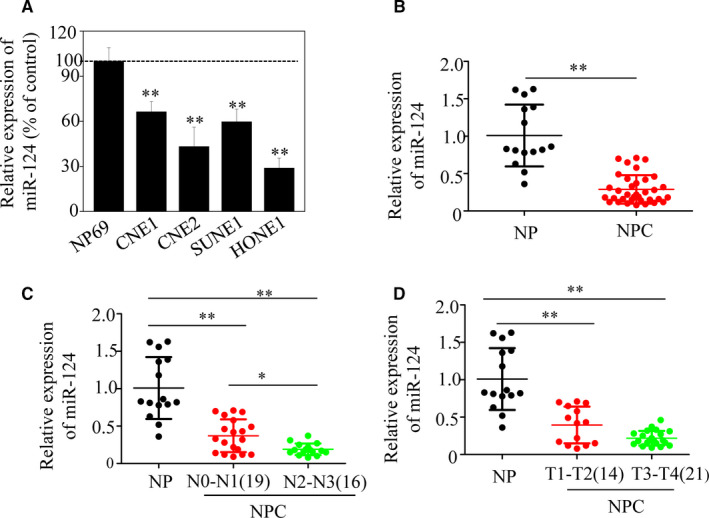
Expression levels of miR‐124 in nasopharyngeal cancer (NPC) cell lines, NPC cancer tissues and non‐cancer nasopharyngitis (NP) tissues. (A) Decreased expression of miR‐124 in NPC cell lines, compared with NP69 cells (*P < .05). (B) Relative miR‐124 expression in NPC tissues determined by qRT‐PCR was reduced by more than 50% compared with NP tissues. Moreover, miR‐124 expression was inversely correlated with lymph node metastasis (C) and T stage (D) in NPC (*P < .05; **P < .01)

To determine the clinical significance of miR‐124 expression in NPC, we first analysed its expression in four NPC cell lines and a non‐cancerous cell line, NP69. By qRT‐PCR, miR‐124 was shown to be significantly down‐regulated in all four NPC cell lines compared with NP69 cells (Figure 1A). Moreover, miR‐124 expression was examined in 35 human primary NPC clinical tissue and 15 NP biopsy samples and average miR‐124 expression was significantly lower in NPC than NP tissues (Figure 1B). Furthermore, miR‐124 expression in patients with NPC was significantly associated with the degree of metastasis to regional lymph nodes and NPC tumour stage of microRNA‐124 expression in patients with N2 and N3 was lower than in those with N0 and N1 stage disease (Figure 1C). Additionally, miR‐124 expression was correlated with T stage (progression), with miR‐124 levels relatively high in T1 and T2 tumours than in T3 and T4 tumours (Figure 1D). There were no significant correlations between miR‐124 expression levels and sex, age or tumour size in patients with NPC (data not shown). These results indicate that reduced miR‐124 expression may be important for NPC initiation and progression.

### 3.2 miR‐124 up‐regulation suppresses the stem‐like phenotype and enhances radiosensitivity of NPC cells

**FIGURE 2:**
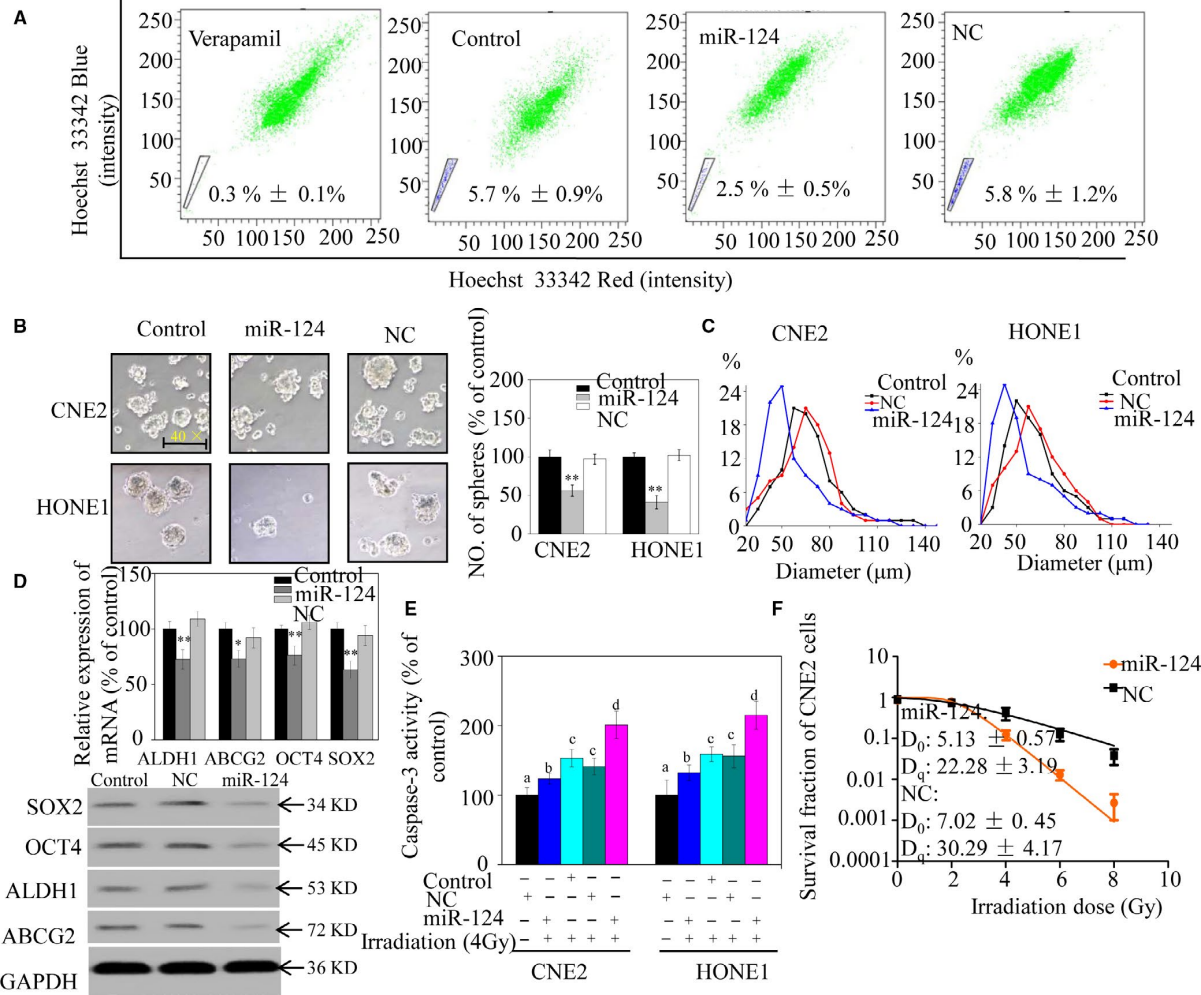
miR‐124 suppresses stem‐like properties and enhances the radiosensitivity of nasopharyngeal carcinoma (NPC) cells in vitro. A, Sorting of side population CNE2 cells using Hoechst 33342. B, Incidence of tumorspheres after 14 days of culture. C, Tumorsphere diameter in CNE2 (left panel) and HONE1 (right panel) cells, presented as frequency distributions (approximately 60 tumorspheres per group were analysed). D, mRNA (upper panel) and protein (lower panel) expression levels in CNE2 cells. E, Caspase‐3 activity in NPC cells after irradiation. Bars with different characters indicate significant differences (*P* < .05). F, Colony‐forming ability of CNE2 cells exposed to various doses of irradiation. The survival fraction (SF) assay was analysed using the single‐hit multi‐target theory formula. Significant differences between the miR‐124 mimic and negative control (NC) groups are indicated by *P < .05 and **P < .01

Side population cells in a human NPC cell line have stem cell characteristics.^4^ Thus, SP cells were sorted to study the functional relevance of miR‐124 in CSCs. The results indicated that the proportion of SP cells in CNE2 cell cultures decreased from 5.8% in control to 2.5% in those transfected with miR‐124 mimic (Figure 2A). CSCs are abundant in non‐adherent spherical cell clusters, termed tumorspheres.^6^ Tumorsphere formation assays showed that miR‐124 inhibited their generation. The number of tumorspheres was 42%‐63% lower, and tumours were also significantly smaller, in cells transfected with miR‐124 mimic, compared with untransfected control cells (Figure 2B,C). Additionally, levels of CSC markers involved in NPC cell stemness, including ABCG2, ALDH1, OCT4 and SOX2, were reduced in miR‐124 mimic–transfected CNE2 cells relative to controls (Figure 2D).

Cancer stem cells are one of the most important factors underlying radiotherapy resistance. We have been suggested that overexpression of miR‐124 could increase NPC cell sensitivity to radiotherapy. To determine whether miR‐124 can increase irradiation‐induced apoptosis of NPC cells, we investigated caspase‐3 activity. Up‐regulation of miR‐124 increased caspase‐3 activity in NPC cells (Figure 2E), whereas colony formation assays indicated that the relative SF of miR‐124 mimic–transfected NPC cells was significantly lower than that of control cells. When dose‐survival curves were fitted, D_0_ and Dq values for miR‐124 mimic–transfected CNE2 cells were both significantly lower than those for control cells (Figure 2F). Hence, caspase‐3 activity and clonogenic assays confirmed that miR‐124 overexpression rendered NPC cells much more sensitive to irradiation than controls. These results show that miR‐124 suppresses NPC stem cell properties in vitro.

### 3.3 miR‐124 down‐regulation promotes stemness and reduces NPC cell radiosensitivity

**FIGURE 3:**
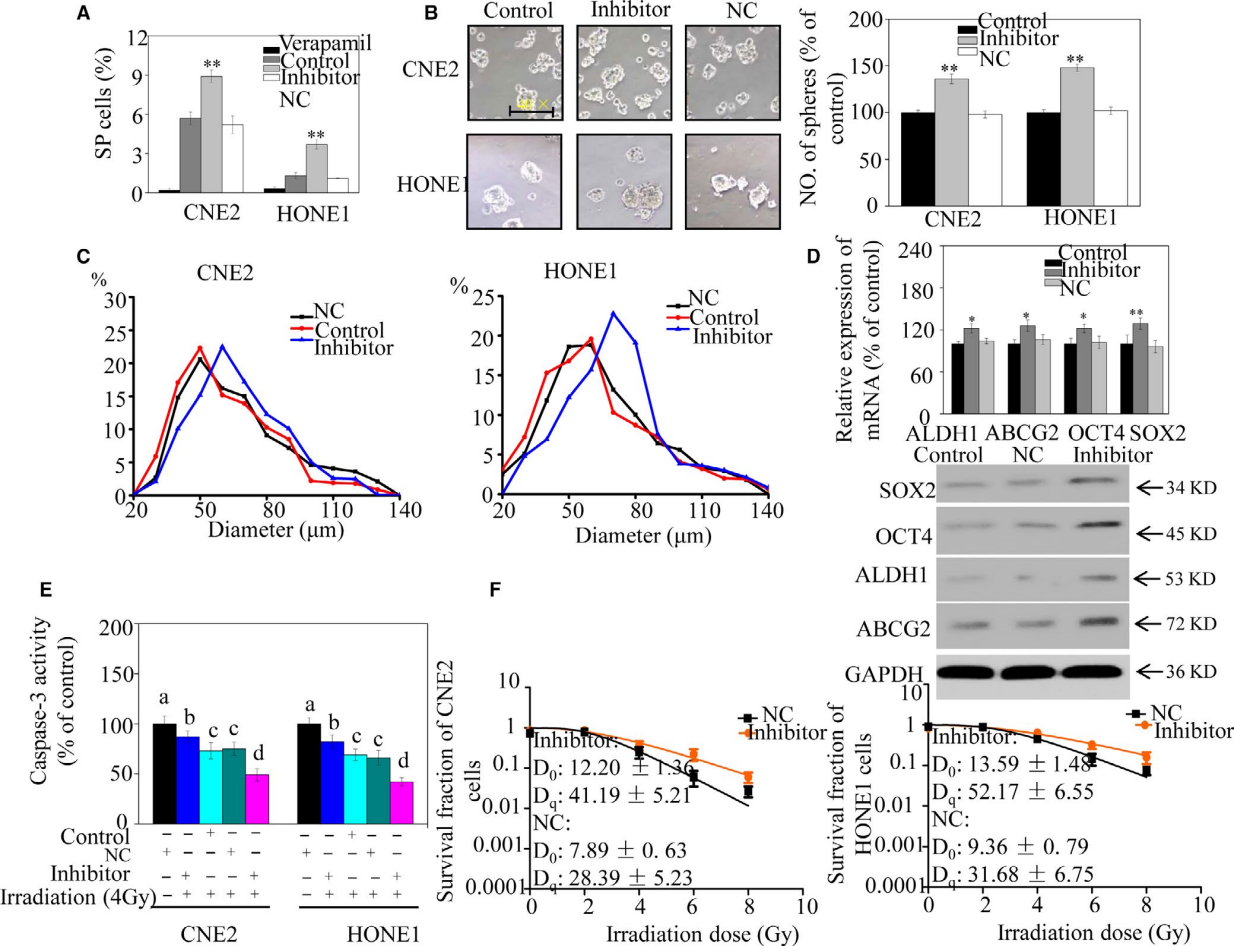
miR‐124 down‐regulation promotes stem‐like properties and reduces radiosensitivity of nasopharyngeal carcinoma (NPC) cells in vitro. A, Sorting of NPC cells transfected with either a miR‐124 inhibitor or a negative control (NC) miRNA using Hoechst 33342. The proportion of side population (SP) cells in NPC cell lines was evaluated. B, Representative images of CNE2 and HONE1 tumorspheres transfected with either an miR‐124 inhibitor or NC (left panel). Number of tumorspheres generated from NPC cells (right panel). C, Diameters of tumorspheres of CNE2 (left panel) and HONE1 cells (right panel), presented as frequency distributions. D, mRNA (upper panel) and protein (lower panel) expression levels of CSC markers in CNE2 cells. E, Caspase‐3 activity of NPC cells transfected with either a miR‐124 inhibitor or NC after irradiation. Bars with different characters indicate significant differences (*P* < .05). F, Colony‐forming ability of NPC cells. The survival fraction for the colony formation assay was analysed. Significant differences between the miR‐124 mimic and NC are indicated by *P < .05 and **P < .01

The effect of miR‐124 on the stem‐like phenotype of NPC cells was further examined. Compared with the control group, significantly more SP cells were present among NPC cells with miR‐124 down‐regulated (Figure 3A). Furthermore, results from tumorsphere formation assays indicated that more and larger spheres were formed in cells with miR‐124 down‐regulated than in controls (Figure 3B,C). In addition, we observed significantly increased expression levels of stem cell markers in CNE2 cells with miR‐124 down‐regulated relative to controls (Figure 3D).

To examine whether miR‐124 increased NPC cell sensitivity to radiotherapy, caspase‐3 activity was measured after cell irradiation. The results indicated that caspase‐3 activity was significantly following irradiation in cells with miR‐124 down‐regulated relative to control cells (Figure 3E). We then assessed survival and proliferation of NPC cell lines using a clonogenic assay. As expected, SFs of the miR‐124 down‐regulated population were significantly higher than those of control cells (particularly with doses ≥4 Gy). D_0_ and Dq values of miR‐124 inhibitor–transfected NPC cells were also both significantly higher than those of control cells (Figure 3F). Hence, caspase‐3 activity and clonogenic assay results support the concept that miR‐124 increases NPC cell sensitivity to radiotherapy. Taken together, these results indicate that miR‐124 inhibition promotes a stem‐like phenotype in NPC cells in vitro.

### 3.4 Expression of JAMA, a direct target of miR‐124, correlates with of NPC progression

**FIGURE 4:**
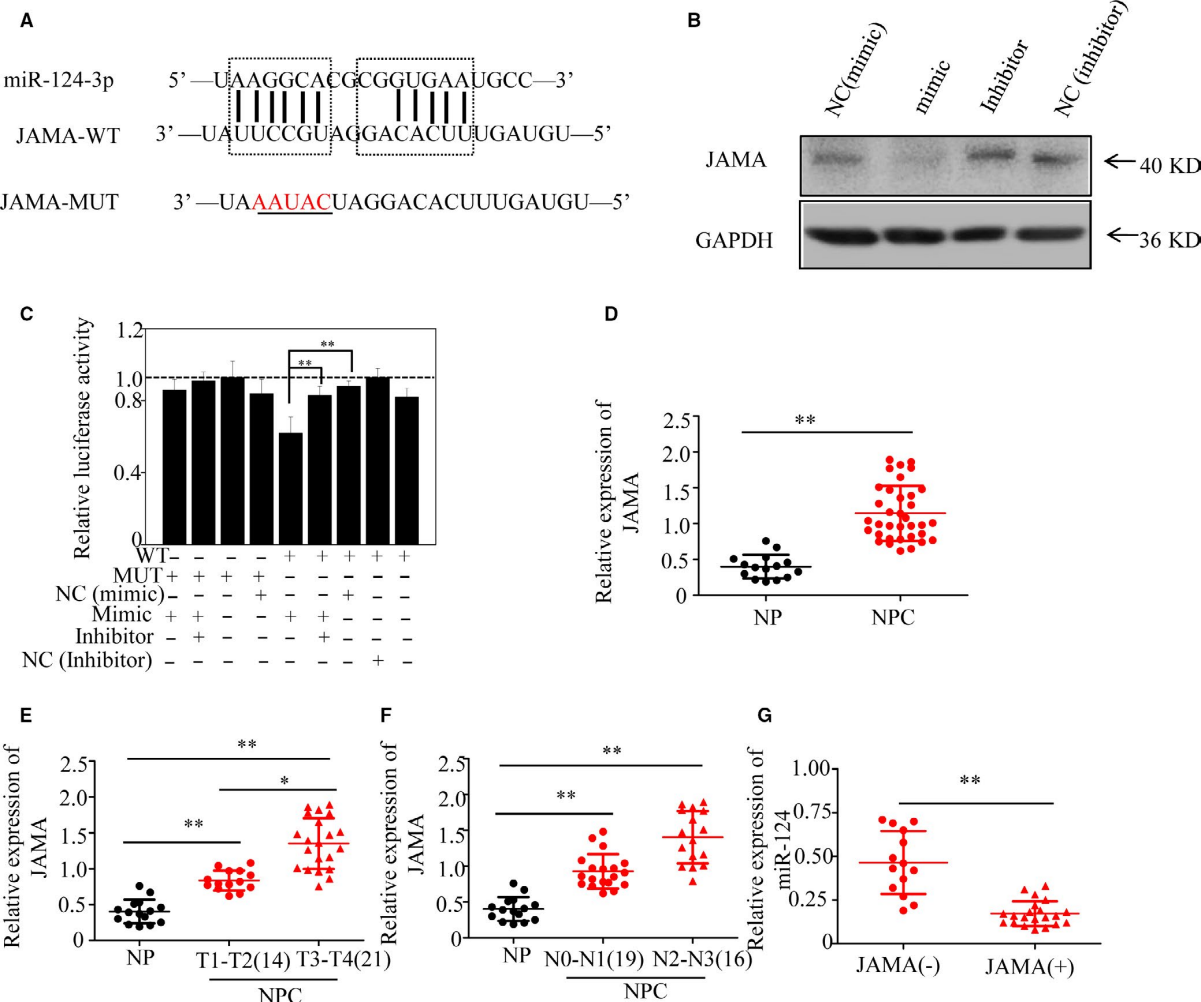
Levels of junctional adhesion molecule A (JAMA), a direct target of miR‐124, correlate with nasopharyngeal carcinoma (NPC) progression. (A) Putative target sites for miR‐124 in the 3′UTR of JAMA. Target sequences in the *JAMA* 3′UTR were mutated. (B) JAMA protein expression levels in CNE2 cell lines were analysed by Western blotting 48 h after transfection with the miR‐124 mimic, inhibitor or negative control (NC). (C) HEK293T cells were cotransfected with miR‐124 mimic, inhibitor or NC and luciferase reporters carrying either the predicted miRNA target site in *JAMA* 3′UTR (WT) or its corresponding mutant (MUT). Data are shown as the mean ± SE. (D–G) Levels of *JAMA* were detected by qRT‐PCR in NPC and non‐cancer nasopharyngitis (NP) tissue specimens, and normalized to those of *GAPDH*. (D) Average *JAMA* expression in 35 cases of NPC and 15 cases of NP. JAMA expression is associated with tumour stage (E) and lymph node metastasis (F) of NPC. (G) miR‐124 expression negatively correlated with JAMA protein expression, as determined by immunohistochemistry in NPC and NP tissues (+ represents score + to +++) (*P < .05 and **P < .01)

To understand the mechanisms underlying the effects of miR‐124 on CSCs, we performed in silico prediction of miR‐124 target genes. A predicted binding site for miR‐124 was detected in the 3'UTR region of JAMA (Figure 4A). Moreover, JAMA protein expression was reduced in CNE2 cells transfected with miR‐124 mimics and significantly increased in cells transfected with an miR‐124 inhibitor (Figure 4B). To determine whether JAMA is a direct target of miR‐124, a double‐luciferase reporter assay was performed. The results indicated that miR‐124 overexpression suppressed luciferase activity from the wild‐type JAMA 3'UTR luciferase construct (Figure 4C), but not from the mutant JAMA 3'UTR. Furthermore, NC miRNA had no influence on luciferase activity from the wild‐type JAMA 3'UTR in HEK293T cells.

To further confirm the relationship between miR‐124 and JAMA, 35 human primary NPC clinical tissue and 15 NP biopsy samples were evaluated for clinicopathological significance. As shown in Figure 4D, JAMA expression was significantly higher in NPC than in NP samples. Further analysis showed that JAMA expression was lower in stage T1‐T2 NPC samples, than T3‐T4 stage NPC samples, showing a significant correlation between JAMA levels and tumour stage (Figure 4E). JAMA expression levels in patients with N2 and N3 were higher than those in patients with N0 and N1 (Figure 4F). Moreover, we observed that down‐regulation of miR‐124 expression correlated with higher JAMA expression in NPC (Figure 4G). Collectively, these data provide strong evidence that high JAMA expression is closely related to NPC progression, and show that JAMA is an authentic target of miR‐124.

### 3.5 Overexpression of JAMA could partially reverse the stem‐like phenotype and reduce radiosensitivity of NPC cells

**FIGURE 5:**
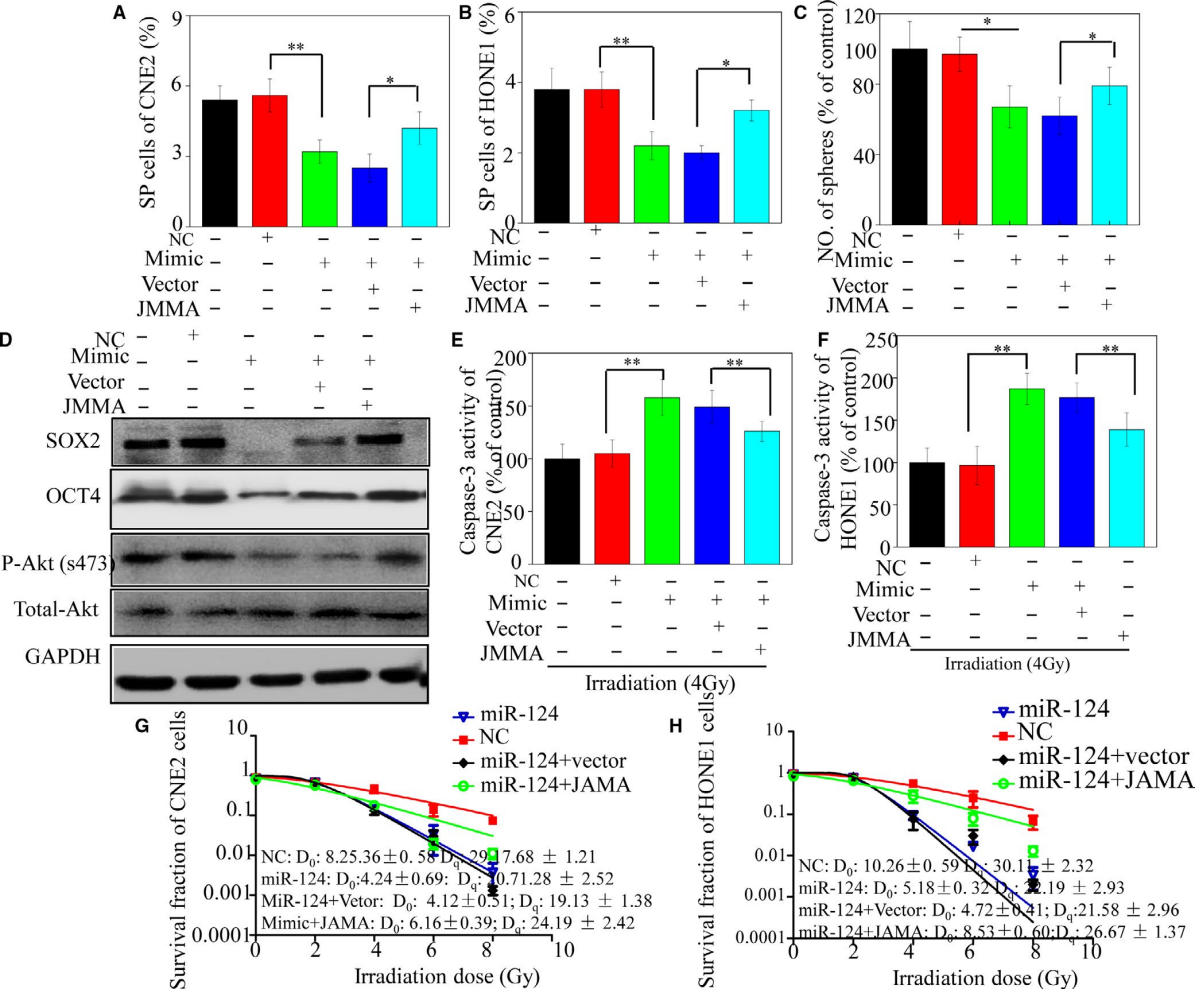
Overexpression of JAMA partially reverses the stem‐like phenotype and reduced radiosensitivity of cancer cells. miR‐124 mimics were transfected into cells using a lentivirus vector expressing JAMA. JAMA restoration significantly increased SP cells in CNE2 (A) and HONE1 (B) cells suppressed by miR‐124. Tumorsphere formation assays indicated that JAMA restoration partially abrogated the inhibitory effect of miR‐124 on tumorsphere formation ability by the CNE2 (C) cell line. (D) Expression levels of OCT4, SOX2 and p‐Akt were detected by Western blotting. JAMA overexpression blocked increased caspase‐3 activity in CNE2 (E) and HONE1 (F) cell lines. After irradiation, JAMA overexpression reduced radiosensitivity of CNE2 (G) and HONE1 (H) cell lines (*P < .05 and **P < .01)

To elucidate whether the stemness‐suppressive effect of miR‐124 was mediated by JAMA in NPC cells, we transfected them with miR‐124 mimics using a lentivirus vector expressing JAMA lacking its 3′UTR. Flow cytometry results indicated that restoring JAMA significantly increased the percentage of SP cells suppressed by miR‐124 (Figure 5A,B). Furthermore, results from tumorsphere formation assays also indicated that cell lines transfected with miR‐124 mimics exhibited a significant decrease in tumorsphere number compared with those transfected with NC miRNA. Conversely, restoring JAMA partially abrogated the inhibitory effect of miR‐124 on CSC tumorsphere formation (Figure 5C). Western blotting data also indicated that stem cell properties were restored by JAMA (Figure 5D). Subsequently, we evaluated whether caspase‐3 activity could be rescued by ectopic expression of JAMA. As expected, increased caspase‐3 activity after irradiation, which was induced by miR‐124 transfection, was partially abrogated by JAMA overexpression (Figure 5E,F). Our previous study indicated that JAMA‐induced EMT may activate the protein kinase B (PKB, also known as Akt) pathway in NPC cells.^17^ Thus, expression of key proteins associated with the Akt pathway and stemness was evaluated in xenograft tumours by Western blotting. The results demonstrated that miR‐124 caused JAMA down‐regulation, and increased levels of phosphorylated Akt and SOX2, indicating that miR‐124–induced inhibition of stem‐like behaviour may be associated with the Akt pathway. Moreover, restoration of JAMA partially reduced the radiosensitivity of CNE2 and HONE1 cell lines, as determined by clonogenic assays (Figure 5G,H). Collectively, these results demonstrate that miR‐124 targets JAMA directly, resulting in suppression of stem cell properties and enhanced radiosensitivity in NPC cells.

### 3.6 Up‐regulation of miR‐124 suppresses NPC cell tumorigenicity in vivo

**FIGURE 6:**
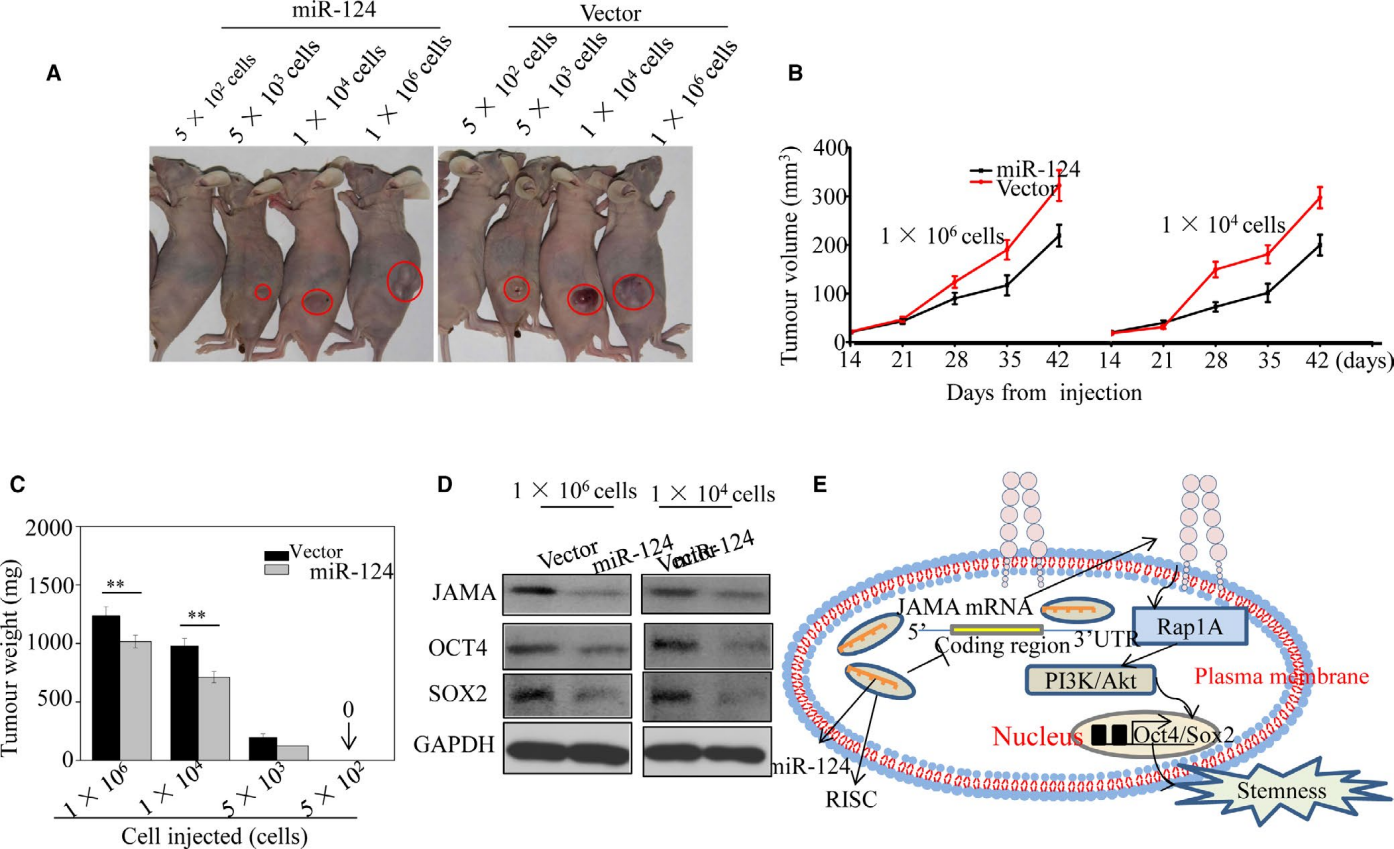
Up‐regulation of miR‐124 suppresses tumorigenicity and enhances radiosensitivity of cancer cells in vivo. A, Representative images of tumours in different groups. B, Growth curves for tumour formation after implantation. C, Histograms show the mean tumour weights of each group. D, Expression of proteins associated with stemness markers, detected in xenograft tumours by Western blotting (*P < .05; **P < .01). E, Proposed signalling pathways involved in miR‐124 inhibition of NPC cell stemness. miR‐124 reduced expression of JAMA by binding to its 3′UTR. Reduced JAMA levels are accompanied by down‐regulation of Akt phosphorylation, a downstream target of SOX2, suggesting that inhibition of stemness by miR‐124 could involve inactivation of the JAMA/Akt pathway

To confirm the effect of miR‐124 on NPC cell stemness, different numbers of CNE2 cells overexpressing miR‐124 or control vector were subcutaneously injected into both posterior flanks of nude mice. As shown in Figure 6A and Table 1, the miR‐124 and vector groups showed similar tumour formation capability when 1 × 10^6^ cells were injected; however, on injection of 1 × 10^4^ cells or fewer, the miR‐124 group exhibited lower tumorigenicity compared with the vector group. Statistical analysis indicated that the miR‐124 group was more able to form tumours than the vector group. These results are consistent with previous reports indicating that CSC and non‐CSCs showed similar tumour‐forming ability on injection of 1 × 10^6^ cells; however, non‐CSCs exhibited lower tumorigenicity than CSCs when less than 1 × 10^4^ cells were injected.^26^, ^27^ In addition, tumours in the miR‐124 group grew more slowly than those in the vector group following implantation of either 1 × 10^6^ or 1 × 10^4^ cells (Figure 6B). Consistent with tumour size, tumour weights in the miR‐124 group were lower than those in the control group (Figure 6C). The expression levels of key proteins associated with stemness were detected in xenograft tumours using Western blotting. In vivo results demonstrated that miR‐124 caused down‐regulation of JAMA, OCT4 and SOX2, indicating that miR‐124 can inhibit stem‐like behaviour (Figure 6D). Based on these results and those of our previous study, we conclude that miR‐124 can inhibit stem‐like properties and enhance radiosensitivity directly by targeting the 3′UTR of *JAMA* via the Akt pathway in NPC cells (Figure 6E).

**TABLE 1:** Tumour formation in BALB/c nude mice initiated by CNE2 cell lines transfected with miR‐124 or control vectors

Cell injected	Tumour incidence/number of injections	
	Vector	JAMA‐shRNA
5 × 10^2^	0/6	0/6
5 × 10^3^	4/6	1/6
1 × 10^4^	5/6	3/6
1 × 10^6^	6/6	6/6
CSC frequency	1 in 5476	1 in 18 511
95% CI	1 in 11 089 to 1 in 2705	1 in 49 226 to 1 in 6961
*P* value	.040	

### 3.7 Reduced miR‐124 expression correlates with inferior prognosis in NPC

**FIGURE 7:**
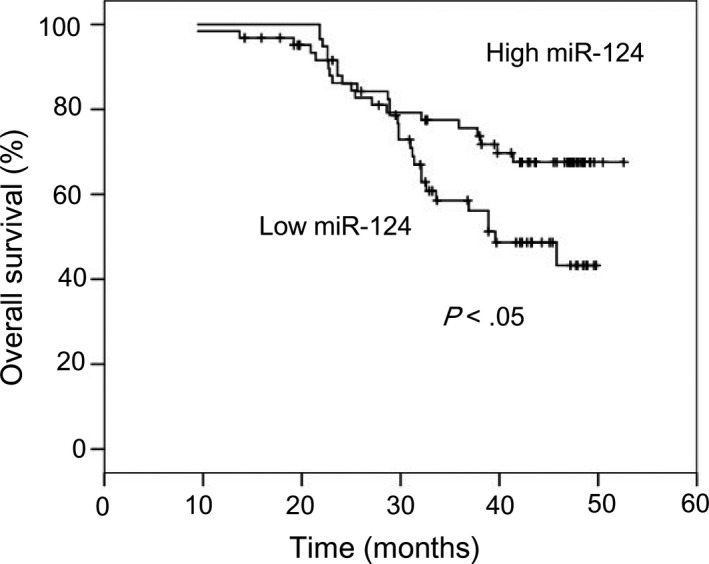
miR‐124 down‐regulation correlates with poor prognosis in NPC. A total of 121 patients with NPC were analysed. Kaplan‐Meier survival curves for NPC were plotted according to miR‐124 expression; differences in survival were evaluated using the log‐rank test

To determine the clinical significance of miR‐124 expression in patients with NPC, its levels were examined in 121 clinical human primary NPC tissues. The results indicated that patients with low miR‐124 expression levels had poorer OS (Figure 7). Cumulative 5‐year OS rates were 67.6% and 43.3% in patients with low and high miR‐483‐5p expression, respectively. In summary, these data suggest that miR‐124 is associated with superior prognosis in NPC.

## 4 DISCUSSION

Nasopharyngeal carcinoma is one of the most common malignancies in south‐eastern China,^1^ and therapy failure in NPC may be due to CSCs.^3^ miR‐124 can mediate stem cell differentiation via targeting various genes,^28^ and further investigation into the role of miR‐124 in NPC CSCs is warranted. In this study, we showed that miR‐124 expression levels were inversely correlated with tumour stage and lymph node metastasis in patients with NPC. Moreover, miR‐124 inhibited NPC stemness both in vitro and in vivo. Mechanistic studies indicated that JAMA was down‐regulated by miR‐124 through its 3'UTR. These findings provide novel insights into the molecular mechanisms underlying failure to respond to treatment in patients with NPC.

miR‐124‐3p (known as miR‐124 or miR‐124a) and miR‐124‐5p (known as miR‐124*) are both mature forms of miR‐124, a brain‐enriched miRNA that has been broadly investigated in the context of physiological neural development. Numerous studies have demonstrated that miR‐124 plays an important role in inhibiting stem‐like traits of cells. For example, Liu et al found that miR‐124 was considerably up‐regulated during HSC differentiation, whereas miR‐124 knockdown slowed HSC differentiation and caused an expansion of haematopoietic progenitor cells in vitro.^29^ Further, Silber et al confirmed that miRNA‐124 induced differentiation of adult mouse neural stem cells, and human glioblastoma multiforme‐derived stem cells.^15^ Moreover, a study by Xia et al demonstrated that miR‐124 inhibits the glioma stem–like traits by targeting Snail 2.^14^ These results are in good agreement with our findings that miR‐124 is a negative regulator of stem‐like traits of NPC cells. Nevertheless, some studies have shown that miR‐124 inhibits myogenic and cardiomyocyte differentiation of mesenchymal stem cells.^30^, ^31^ The different effects of miR‐124 on the stem‐like traits of NPC cells compared with mesenchymal stem cells could be dependent on disease, cells and surroundings.^32^ In summary, we have demonstrated for the first time that miR‐124 plays an important role in inhibiting the stem‐like properties of NPC cells.

Down‐regulation of miR‐124 in various cancers plays a vital role in tumorigenesis and tumour progression. Han et al showed that ectopic expression of miR‐124 in breast cancer cell lines strongly inhibited cell motility and invasive capacity via targeting CD151.^33^ Further, Zhou et al confirmed that miR‐124 inhibited cell proliferation, invasion and metastasis by interacting with the 3′UTR of Rho‐associated protein kinase 1 (ROCK1), and was significantly associated with better colorectal cancer survival rates.^34^ Moreover, Li et al demonstrated that miR‐124 inhibited lung cancer cell growth and migration via direct binding to the 3'UTR of *CDH2*, whereas down‐regulation of miR‐124 was also associated with higher tumour stage and levels of lymph node metastasis.^35^ In this study, we demonstrate for the first time that miR‐124 is significantly down‐regulated in NPC compared with NP, as well as inversely associated with advanced tumour stage and lymph node metastasis. Moreover, miR‐124 could suppress the stem‐like phenotype and increase sensitivity of NPC cells to radiotherapy, supporting its potential as a tumour suppressor in NPC.

In the present study, we identified JAMA as a novel target of miR‐124. Our results suggested that miR‐124 directly targeted JAMA by binding to its 3′UTR, resulting in significantly decreased JAMA protein expression. To our best knowledge, these observations provide the first line of evidence that miR‐124 acts as a repressor of JAMA, a cell adhesion molecule found on the surface of human platelets, which functions in cell migration and proliferation^18^, ^19^; however, the role of JAMA in regulating NPC stem–like behaviour is unknown. Here, we found that JAMA up‐regulation can enhance the stem‐like properties of NPC cells in vitro and in vivo. Previous studies support this finding, as cancer cells that undergo EMT are capable of generating cells with stem cell properties, which are more efficient at forming mammospheres, soft agar colonies and tumours.^36^ Moreover, we previously demonstrated that JAMA can induce EMT of NPCs.^17^ Therefore, JAMA may promote generation of CSCs from other more differentiated neoplastic cells. Furthermore, JAMA is expressed in both murine and zebrafish HSC fractions. Stemness behaviour in JAMA‐positive cells is greater than that in JAMA‐negative cells in the enriched HSC fraction in these tissues,^20^ whereas Kobayashi et al showed that JAMA regulates HSC fate through Notch signalling in zebrafish. Loss of JAMA function results in reduction in HSCs.^37^ These studies show that JAMA can regulate HSC stemness behaviour, strongly supporting its ability to enhance stem cell properties of NPC cells in vitro when up‐regulated. This is the first report to demonstrate that JAMA is associated with CSC‐like phenotypes in cancer cells, particularly in NPCs.

In conclusion, we found that down‐regulation of miR‐124 in patients with NPC was inversely associated with advanced tumour stage and positive lymph node metastasis. miRNA‐124 inhibits NPC stem‐like properties *via* direct targeting of the *JAMA* 3′UTR. These findings provide novel insights into the molecular mechanisms underlying failure to respond to treatment in patients with NPC. As such, restoring miR‐124 activity represents a promising strategy for NPC therapy.

## CONFLICT OF INTERESTS

No conflict of interests exists.

## AUTHORS' CONTRIBUTIONS

YY, TY(h) and ZM participated in the conception and design of the study and the critical revision of the manuscript for important intellectual content. TY(m), TY(h), TY and JL performed the experiments and data analysis. AM, SJ, ZX, TY and ZR interpreted the data and produced the draft of the manuscript. TY(h) obtained funding for the study. All authors have read and approved the manuscript.

## ETHICS APPROVAL AND CONSENT TO PARTICIPATE

All animal experiments and clinical sample collection were approved by the medical ethics committee of the Affiliated Cancer Hospital & Institute of Guangzhou Medical University (Guangzhou). All patients involved in this study signed an informed consent form.

## CONSENT FOR PUBLICATION

The author grants the publisher the sole and exclusive licence of the full copyright in the contribution, which license the publisher hereby accepts. The publisher shall have the exclusive right throughout the world to publish and sell the contribution in all languages, without limitation.

## PUBLISHER'S NOTE

Springer Nature remains neutral with regard to jurisdictional claims in published maps and institutional affiliations.

## Data Availability

Materials and data are available upon request to the corresponding author.
